# How do the type of QTL effect and the form of the residual term influence QTL detection in multi-parent populations? A case study in the maize EU-NAM population

**DOI:** 10.1007/s00122-017-2923-3

**Published:** 2017-05-25

**Authors:** Vincent Garin, Valentin Wimmer, Sofiane Mezmouk, Marcos Malosetti, Fred van Eeuwijk

**Affiliations:** 1 Biometris, Wageningen University and Research Center, P.O Box 100, 6700 AC Wageningen, The Netherlands; 2 KWS SAAT AG, Einbeck, Germany

## Abstract

*****Key message***:**

**In the QTL analysis of multi-parent populations, the inclusion of QTLs with various types of effects can lead to a better description of the phenotypic variation and increased power.**

**Abstract:**

For the type of QTL effect in QTL models for multi-parent populations (MPPs), various options exist to define them with respect to their origin. They can be modelled as referring to close parental lines or to further away ancestral founder lines. QTL models for MPPs can also be characterized by the homo- or heterogeneity of variance for polygenic effects. The most suitable model for the origin of the QTL effect and the homo- or heterogeneity of polygenic effects may be a function of the genetic distance distribution between the parents of MPPs. We investigated the statistical properties of various QTL detection models for MPPs taking into account the genetic distances between the parents of the MPP. We evaluated models with different assumptions about the QTL effect and the form of the residual term using cross validation. For the EU-NAM data, we showed that it can be useful to mix in the same model QTLs with different types of effects (parental, ancestral, or bi-allelic). The benefit of using cross-specific residual terms to handle the heterogeneity of variance was less obvious for this particular data set.

**Electronic supplementary material:**

The online version of this article (doi:10.1007/s00122-017-2923-3) contains supplementary material, which is available to authorized users.

## Introduction

The papers by Rebaï and Goffinet (1993) and Muranty (1996) are early examples of quantitative trait locus (QTL) detection with populations derived from more than two parents. More recently, QTL mapping using multi-parent populations (MPPs) has increased in popularity, where these MPPs include nested association mapping populations (NAM) (McMullen et al. 2009), diallels (Blanc et al. 2006) and factorial designs (Bardol et al. 2013), as well as more complicated MPPs created by intercrossing multiple founders followed by inbreeding, such as in multi-parent advanced generation inter-cross (MAGIC) populations (Cavanagh et al. 2008). Here, we consider MPPs as a collection of crosses between at least three different parents and focus on an NAM population which involves crosses between a central parent and a set of peripheral ones. An MPP QTL analysis would, therefore, be the joint analysis of such a population using a common marker map. Other authors have sometimes called it family mapping (Würschum 2012), combined cross analysis (Li et al. 2005) or multiple-cross analysis (Jourjon et al. 2005).

To structure our reasoning, we present some assumptions on the genetic properties of specific MPPs and make plausible how these properties can affect the choice of a statistical model for QTL mapping. In MPPs, the use of more than two parents potentially increases the allelic diversity in the MPP as a whole and so, increases the chance of segregation at any particular genomic position (Xu 1998). MPPs allow to test genetic effects within different backgrounds (Blanc et al. 2006) and so extend the statistical inference space for the QTL effects (Xie et al. 1998). If the addition of parental genotypes does not increase the allelic diversity at a particular locus, the use of an MPP can still be advantageous with respect to a bi-parental cross, because QTLs representing the same ancestral locus will benefit from an increased sample size to estimate their effects (Li et al. 2005).

The allelic diversity of the population may be related to the genetic distance among the parents of the population. When parents are genetically distant, the expected number of segregating alleles at a particular locus increases with the probability that these alleles are unique to a parental line. On the other hand, when the parents are genetically closer, one expects a reduced number of alleles segregating at a particular locus and that the alleles are shared throughout the population.

It seems beneficial to QTL detection and estimation that the statistical model for the phenotypic variation in an MPP takes into account the genetic properties (number of alleles and diversity) of the MPP. The genetic diversity contained in the MPP can be translated to properties of the statistical model via the form of the QTL effects and the structure of the polygenic variation. This latter variation is the natural variation against which to test QTL effects and determines statistical quantities, such as power and false-positive rate.

*Models for the QTL effect* If the number of segregating alleles at a particular QTL increases (e.g., in a diverse MPP), the statistical model can capture that diversity by allowing more parameters for the QTL effect. In crossing schemes starting from pure lines, it implies estimating a maximum of one effect per parental line (parental model). On the other hand, when MPP genetic diversity is lower, parental relatedness can be used to infer a reduced number of ancestral segregating alleles that need to be estimated, thereby increasing model parsimony and probably also QTL detection power (ancestral model). The lower bound will be reached when only two alleles are segregating in the totality of the MPP (bi-allelic model). Within fixed QTL effect models, the reduction of QTL parameters to be estimated to improve QTL detection power has been a central objective (Rebaï and Goffinet 1993; Jansen et al. 2003; Blanc et al. 2006; Leroux et al. 2014).

The assumption concerning the number of alleles at a QTL position can also be seen from a pedigree or historical perspective. Indeed, when parental QTL effects are appropriate, it implies that the allele origin is more recent than that of ancestral QTLs common to several parents. One can also argue that bi-allelic QTL effects are closer to the original mutation (Powell et al. 2010). These different assumptions about the number of alleles correspond to different ways of modelling genetic relatedness between lines at the QTL position. So far, QTL studies used models that assumed a single type of QTL effect or allele origin (e.g., Blanc et al. 2006 or Würschum et al. 2012). We can, however, imagine that allele origin and the number of alleles segregating at a QTL position can vary along the genome. Therefore, in the present article, we will compare QTL models assuming a single type of genetic relatedness along the genome with a model that allows different types of allele origin.

*Models for the polygenic term* The genetic relationship between the parents of an MPP will also have an influence on the magnitude and structure of the polygenic or residual genetic term. The more diverse the population is, the more heterogeneous the residual variance is expected to be. The heterogeneity of the residual genetic variance may depend on the level of genetic relatedness between the parents of an MPP. Differences in genetic relatedness between pairs of parents can induce different levels of polygenic effect variation, inducing heterogeneity in residual genetic variance. Several studies of QTL mapping in MPPs applied linear models assuming a homogeneous variance for the residual genetic term (Li et al. 2005; Blanc et al. 2006; Yu et al. 2008). Depending on the particular MPP, this assumption might be unrealistic affecting the statistical test used to detect QTLs. To handle heterogeneous variances, some authors used transformed phenotypic data (Walling et al. 2000; Li et al. 2005; Guo et al. 2006). Others, such as Xu (1998), proposed to fit the QTL model by iteratively re-weighted least-squares. Polygenic effects can also be directly modelled for heterogeneity of variance in mixed models (Xu and Atchley 1995; Yu et al. 2006; Wei and Xu 2015). In our study, we alleviated the restriction of classical linear models of homogeneous polygenic variance using models with cross-specific variances for the residual genetic term.

We summarize our expectations for QTL detection in MPPs by the following propositions. We will refer to them to guide the discussion of the results.

### **Proposition 1**

*Models assuming common effects across the population, such as the ancestral or the bi-allelic models, should perform relatively better in MPPs with a narrower genetic basis than in genetically diverse MPPs, since the probability of shared polymorphism is higher in the former than in the latter. In diverse populations, however, the opposite is expected, requiring models with more QTL effect terms to capture the allelic diversity.*

### **Proposition 2**

*The use of different types of QTL effects corresponding to different origins of the QTL allele at different positions along the genome should give a more adequate description of the phenotypic variation with increased QTL detection power.*

### **Proposition 3**

*MPP genetic diversity should be reflected in the genetic variance of the crosses composing the population. Diverse MPPs present potentially more heterogeneity of the within cross genetic variance than less diverse populations. In diverse population, the use of cross-specific residual terms should give a better description of the data than a homogeneous residual term model. In more homogeneous populations, the difference between cross-specific residual terms and homogeneous residual term should be minor.*

In our paper, we evaluate various models for QTL detection in MPPs with different types of QTL effects and residual genetic terms. Beyond the currently existing methods, we propose a multi-QTL effect (MQE) model that allows various types of QTL effects at individual loci, where loci can differ in the most suitable type of QTL effect. We also relax the assumption of constant variance for the residual genetic term using a cross-specific residual term (CSRT) model. We performed QTL detection in three subsets of the EU-NAM Dent population characterized by different degrees of genetic relatedness between the parents. The different models were evaluated using cross validation (CV).

## Materials and methods

To ensure the transparency and the reproducibility of our research, all data files, scripts, and required software can be found in the following repository https://github.com/vincentgarin/MPP_EUNAM. This material makes it possible to reproduce all steps of the analysis, tables, and figures of the article and of the supplemental material. To test the various models, we used the maize EU-NAM Dent panel (Bauer et al. 2013) and formed subsets.

### Genotypic data

The Dent panel of the EU-NAM population was composed of double haploid (DH) lines originating from ten crosses between the central line F353 and ten peripheral parents. This population was developed to represent the maize diversity in Northern Europe and also included the central parent of the US-NAM population, and it has been described in detail in Bauer et al. (2013) and Lehermeier et al. (2014). The offspring lines and the 11 parental lines were genotyped with the Illumina MaizeSNP50 BeadChip containing 56,110 single nucleotide polymorphisms (SNPs) (Ganal et al. 2011). Raw genotypic data were obtained from: http://www.ncbi.nlm.nih.gov/geo/query/acc.cgi?acc=GSE50558. We used the consensus map calculated by Giraud et al. (2014) available at: http://maizegdb.org/data_center/reference?id=9024747. From the original list of markers, we selected the Panzea markers to avoid ascertainment bias (Bustos-Korts et al. 2016).

### Population subsets

**Table 1:** EU-NAM population crosses and simple matching coefficient (SM) between the central (F353) and peripheral parents

Cross	Parent	SM	DMY			PH			Short	Het.	Long
			$\bar{X}$	$\sigma _{g}^{2}$	$h^{2}$	$\bar{X}$	$\sigma _{g}^{2}$	$h^{2}$			
CFD11	UH304	0.761	192.5	47.8	59.4	288.5	37.4	68.9	60	65	0
CFD06	F252	0.633	178.1	120.9	78.0	285.7	65.9	79.9	76	0	0
CFD04	D09	0.618	187.4	31.3	41.9	284.5	67.3	86.5	78	85	0
CFD07	F618	0.589	194.7	20.2	31.2	291.4	45.0	79.5	79	0	0
CFD03	D06	0.586	189.9	78.2	70.9	292.2	52.8	73.8	68	90	0
CFD10	UH250	0.575	187.5	67.6	61.3	287.8	65.5	85.3	0	0	94
CFD09	Mo17	0.567	184.5	88.9	52.3	292.9	58.5	75.8	0	0	53
CFD12	W117	0.565	176.2	75.5	60.4	273.9	102.5	84.6	0	68	84
CFD05	EC169	0.558	184.2	46.5	56.4	283.5	64.8	84.7	0	0	66
CFD02	B73	0.557	193.2	95.6	64.1	294.8	60.8	81.1	0	53	64
Total									361	361	361

Average adjusted mean values ($\bar{X}$), genetic variance components ($\sigma _{g}^{2}$) and within cross heritability ($h^{2}$) for dry matter yield (DMY) and plant height (PH), and number of sampled lines per cross in the different subsets (short, heterogeneous, long)

In the absence of pedigree information, genetic relatedness between parental lines can be estimated by similarity of molecular marker scores. We used the genetic similarity coefficient defined by Nei and Li (1979) (Supplemental file S1), which in our situation corresponds to the simple matching (SM) coefficient. We composed different subsets of the EU-NAM population (Table 1) involving parents with different levels of genetic relatedness between the central and the peripheral parents (see matrix of pairwise SM and PC—supplemental Table S2 and Figure S3). We formed: (1) a “short” subset with the five parents closest to the central parent; (2) a “long” subset with the five most distant parents from the central parent; and (3) a “heterogeneous” subset with a mixture of distant and close parental lines. The average SM between pairs of parents in the subsets decreased from the short to long subsets. $\bar{\mathrm{SM}}$ is equal to 0.639, 0.613, and 0.573 for the short, heterogeneous, and long subsets, respectively.

To assure that all subsets were of equal size, we randomly selected 361 lines from the crosses of the short and heterogeneous subsets to make their size equal to that of the long subset. For QTL analyses, we removed markers that were not segregating in any of the MPPs and that showed a minor allele frequency <0.01 or missing values >10% across the entire MPP. When multiple SNPs mapped at a single chromosome position, we selected the most polymorphic locus. After pre-processing, 5737, 5934, and 6212 SNPs were used for the short, heterogeneous, and long subsets, respectively, 3348 of which were common between the MPPs (see genetic maps—supplemental Figure S4).

### Phenotypic data

We used the raw phenotypic data provided by Lehermeier et al. (2014) http://www.genetics.org/content/198/1/3/suppl/DC1 and calculated the adjusted means and variance components and heritability following their procedure (Table 1). Christina Lehermeier kindly communicated to us the list of genotypes used in her study which allowed us to use the same lines as in Giraud et al. (2014) and Lehermeier et al. (2014). We selected the traits with the lowest and highest average heritability over all crosses: biomass dry matter yield (DMY, decitons per hectare, $\frac{dt}{ha}$, $\bar{h^{2}}=57\%$) at the whole plant level and plant height (PH, cm, $\bar{h^{2}}=81\%$).

### Statistical methodology

Let us start with the general single locus model for an MPP following the notation of Rebaï and Goffinet (1993):

1 $$\begin{aligned} y_{ijk} = \mu _{ij} + \alpha _{i} + \alpha _{j} + g_{ij} + e_{ijk} \end{aligned}$$

where $y_{ijk}$ represents the phenotypic adjusted mean for the *k*th individual from the cross between parents *i* and *j*. $\mu _{ij}$ is the cross mean and $\alpha _{i}$ and $\alpha _{j}$ represent the additive effects associated with the QTL alleles coming from parent *i* and *j*, respectively. $g_{ij}$ is the random polygenic effect due to QTLs elsewhere in the genome with distribution $N(0,\sigma _{g}^{2})$. Finally, $e_{ijk}$ represents the random micro-environmental effect (plot error) having distribution $N(0,\sigma _{e}^{2})$.

Model 1 can be rewritten in matrix notation:

2 $$\begin{aligned} \varvec{y} = \varvec{X}\varvec{\beta } + \varvec{r} \end{aligned}$$

where $\varvec{y}$ is the $[N \times 1]$ vector of phenotypic values. $\varvec{X=[X_{\mathrm{c}}|X_{Q}]}$ is the fixed effect incidence matrix and $\varvec{\beta }' = [\varvec{\beta _{\mathrm{c}}}'|\varvec{\beta _{Q}}']$ is the vector of cross intercepts and QTL effects. $\varvec{X}$ is composed of a part that links observations to the particular cross it belongs to ($\varvec{X_{\mathrm{c}}}$ an $[N \times n_{\mathrm{c}}]$ matrix with $n_{\mathrm{c}}$ representing the number of crosses) and $\varvec{X_{Q}}$ is the part related to the QTL effects. $\varvec{X_{Q}}$ is a matrix of dimensions $[N \times n_{\mathrm{al}}]$ with $n_{\mathrm{al}}$ the number of QTL alleles that are assumed to segregate for the particular QTL locus. The individual elements of $\varvec{X_{Q}}$ take values between 0 and 2 and represent the number of allele copies received by genotype *n* at locus *m*. The number of columns $n_{\mathrm{al}}$ varies with the number of alleles assumed at the QTL position. We propose three models.

*Parental model* This first model assumes that each parent contributes a unique allele to the MPP. In an NAM population, peripheral parents are used only once, so that their QTL effects are nested within the crosses between central and peripheral parents. In the parental model, the individual elements of $\varvec{X_{Q}}$ are the expected numbers of QTL alleles received from the parents given the genotypes of the flanking markers, which were estimated using identity by descent (IBD) probabilities computed with the calc.genoprob() function from the R package qtl (Broman et al. 2003). The parental model corresponds to the connected model in Blanc et al. (2006).

**Fig. 1:**
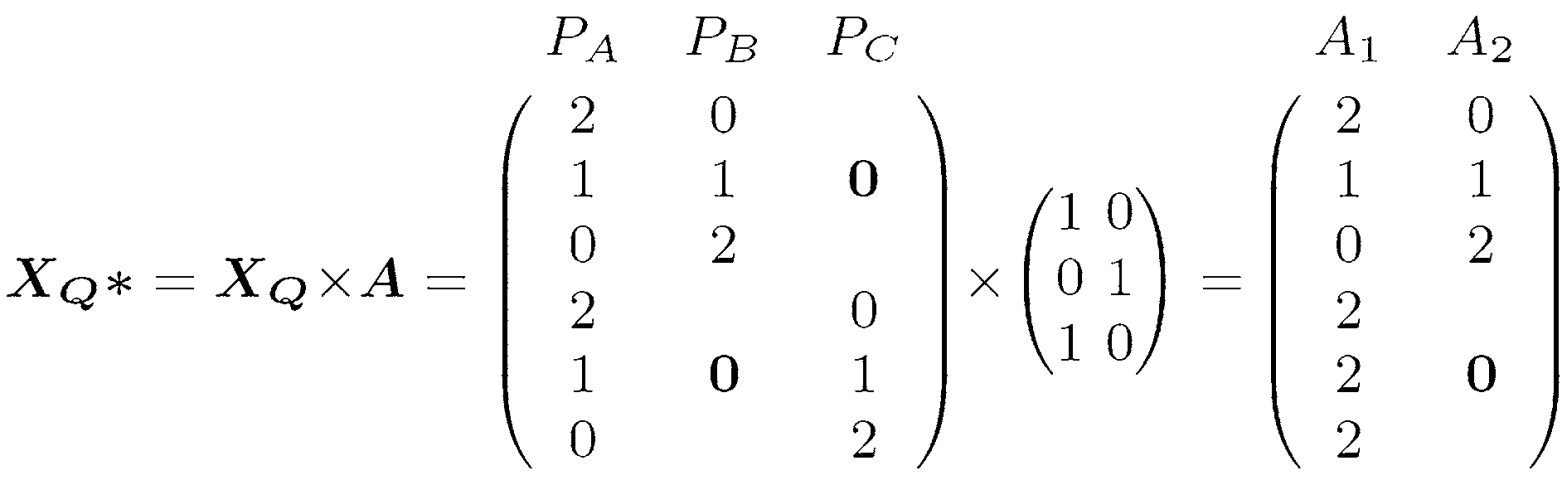
Example of ancestral QTL incidence matrix formation. Parental matrix $\varvec{X_{Q}}$ is transformed by ancestral matrix $\varvec{A}$. Let us assume two crosses with a shared central parent: cross 1 ($P_{A} \times P_{B}$) and cross 2 ($P_{A} \times P_{C}$). Parents A and C are related to the same ancestral source

*Ancestral model* A second option uses relatedness between parents to cluster them into a reduced number of ancestral groups, so $n_{\mathrm{al}} \le P$. Under this model, parents belonging to the same cluster are assumed to transmit the same allele (Jansen et al. 2003; Leroux et al. 2014). For our analyses, the clustering of the parental lines was done at each marker position using a 2 cM window around the position with the R package clusthaplo (Leroux et al. 2014). The grouping is a function of the local similarity score defined by Li and Jiang (2005) and a global similarity defined by kinship coefficients. The results were stored in an ancestral matrix $\varvec{A}$ that allows to modify IBD relationship of the parental model to account for ancestral relatedness (Fig. 1). The ancestral model uses, therefore, both IBD and parental marker score information. This model corresponds to linkage disequilibrium linkage analysis (LDLA) models in Bardol et al. (2013) and Giraud et al. (2014).

*Bi-allelic model* The simplest model assumes that genotypes with the same SNP score transmit the same allele. Genetic relatedness is, therefore, defined based on marker identity by state (IBS) information. In this model, $\varvec{X_{Q}}$ becomes a vector with values 0, 1, or 2 corresponding to the number of copies of the least frequent allele. For the bi-allelic model, missing marker genotypes were imputed by the software package Beagle (Browning and Browning 2013) via the synbreed R package (Wimmer et al. 2012). This model is used in genome-wide association studies (GWAS), and corresponds to model B in Würschum et al. (2012) and the association mapping model in Liu et al. (2012).

The Wald test was used to test the global null hypothesis of all allele QTL effects equal to 0 (McCulloch and Searle 2001, 5.39). The choice between the three models can be seen as a search for an optimum between parsimony and goodness of fit. If the allelic series are complex, such as in a diverse population, then the parental model will be more suitable. On the other hand, if QTL effects are shared through the population, then the ancestral or the bi-allelic model will allow to gain in power by estimating a reduced number of parameters (for more detail, see supplemental file S5).

If cofactors are included when searching for QTLs, $\varvec{X}$ is augmented to $\varvec{[X_{\mathrm{c}}|X_{q}|X_{Q}]}$, where $\varvec{X_{q}}$ is the cofactor incidence matrix and $\varvec{\beta }' = [\varvec{\beta _{\mathrm{c}}}'|\varvec{\beta _{q}}'|\varvec{\beta _{Q}}']$ is the vector of fixed effects, with $\varvec{\beta _{q}}'$ representing the cofactors’ effects. In all our models, the QTL genetic effects were estimated by setting the most frequent allele as a reference. In the parental and ancestral models, it corresponded to the central parent F353 or the ancestral group containing F353.

*Multi-QTL effect (MQE) model* The parental, ancestral, and bi-allelic models have already been used in other papers (e.g., Bardol et al. 2013 or Giraud et al. 2014). These studies restricted the model to a single type of QTL effect, keeping the same type of incidence matrix across all loci. However, allelic effects in an MPP may vary across loci (Bardol et al. 2013), so a more flexible modelling approach would allow the incidence matrix to change from locus to locus. As an alternative, we propose a procedure to build multi-QTL effect models in which different loci can be modelled by different types of QTL effect (parental, ancestral, or bi-allelic).

For the residual term $\varvec{r}$ in Model 2, we propose two models concerning the residual variance $\varvec{R}$. The simplest model assumes constant variance (homogeneous variance residual term, HRT) $\varvec{R}=\varvec{I}\sigma _{r}^{2}$. This is the model used for the residual polygenic and environmental variances in the original paper by (Rebaï and Goffinet 1993). A different model allows cross-specific variance residual terms (CSRT), which is more appropriate when heterogeneous polygenic effects are expected due to heterogeneous genetic distances among parents. In this case, $\varvec{R} = \bigoplus _{c=1}^{n_{\mathrm{c}}}\sigma _{r_{\mathrm{c}}}^{2}$, where $c= 1,\ldots ,n_{c}$ is the cross index. This model is similar to the one used by Xu (1998). From a theoretical perspective, the use of a single residual genetic variance component will lead to under and overestimation of this variance, depending on the cross, and, therefore, to an increase of the number of false positives and false negatives when heterogeneity of the polygenic effect is indeed high (for more detail, see supplemental file S5).

### Fast CSRT model

The estimation of an exact solution for the CSRT model at each marker position during the CV procedure is computationally too demanding. Therefore, we propose a fast CSRT (f-CSRT) algorithm to compute approximate solutions. To calculate such an approximation, we estimated first the residual term $\varvec{R}$ in a model without QTL term and then used it in the Wald test to estimate the significance of the QTL effects along the genome (for more details, see supplemental file S6).

### QTL detection procedure

The combination of the four QTL effects (parental, ancestral, bi-allelic, and MQE) with the two residual terms (HRT and CSRT) gives eight possible models for QTL detection. For the parental, ancestral, and bi-allelic HRT models, the significance thresholds were determined by 1000 genome-wide permutations taking the $-\mathrm{log}_{10}(p$ value) of the upper 95$\%$ Wald statistic under the empirical null distribution as the critical value for rejection (Churchill and Doerge 1994). The determination significance thresholds for the CSRT models were computationally too demanding. Therefore, we used the same threshold values as the one of the corresponding HRT model. The significance thresholds of the MQE models were obtained by averaging the thresholds of parental, ancestral, and bi-allelic models.

For the QTL detection methods based on QTL models with a single effect, a first run of simple interval mapping (SIM) was followed by two runs of composite interval mapping (CIM) by adding markers as cofactors (Zeng 1993, 1994). We took care that QTLs (and cofactors) were spaced by a minimum distance of 20 cM. A multi-QTL model was created from the full list of QTLs detected after CIM by a backward elimination procedure with confidence level set at $\alpha = 0.01$. We used the same procedure as Han et al. (2016) to compute the proportion of genetic variance explained by the QTLs in the training set (TS): $\mathrm{pTS} = R_{\mathrm{adj}}^{2}/h^{2}$. For the HRT model, we used $R_{\mathrm{adj}}^{2} = 1 - \frac{\mathrm{RSS}_{\mathrm{full}}/df_{\mathrm{full}}}{\mathrm{RSS}_{\mathrm{red}}/df_{\mathrm{red}}}$. $\mathrm{RSS}_{\mathrm{full}}$ and $df_{\mathrm{full}}$ are the residual sum of squares and degree of freedom of a model including QTLs, while $\mathrm{RSS}_{\mathrm{red}}$ and $df_{\mathrm{red}}$ come from a model without QTLs. Note that both models contained a cross-specific intercept term that removes the between cross variation.

For the CSRT model, we used the likelihood $R^{2}$ defined by Cox and Snell (1989): $R_{\mathrm{LR}}^{2} = 1 - \mathrm{exp}(-\frac{2}{n}(\mathrm{log}L_{\mathrm{full}} - \mathrm{log}L_{\mathrm{red}}))$, where $L_{\mathrm{full}}$ and $L_{\mathrm{red}}$ represent the likelihood statistic of the full and reduced models, respectively. For the CSRT model, following the recommendations of Sun et al. (2010), we estimated the likelihood of the reduced and full model using maximum likelihood estimation (not REML). We adjusted the likelihood $R^{2}$ using formula 2 from Utz et al. (2000): $R_{\mathrm{adj}}^{2} = R^{2} - [(\frac{df_{\mathrm{QTL}}}{df_{\mathrm{full}}})\times (1-R^{2})]$.

To build the multi-locus MQE models, we used a forward selection approach, where at each step, a new QTL was added that was allowed to have either a parental, an ancestral, or a bi-allelic effect. To identify a new QTL, we computed three genome-wide profiles using the same type of QTL effect for the tested position (parental, ancestral, or bi-allelic). Then, we selected in each profile the most significant position based on the $-\mathrm{log}_{10}(p$ value) with its type of QTL effect. From these candidate positions (and effects), we selected the one that increased the most the model $R_{\mathrm{adj}}^{2}$. The selected position with its type of QTL effect entered the model, and the process was repeated until no more significant positions could be added (for more detail, see supplemental file S7).

HRT models were fitted by least-squares (lm() function in R), and CSRT by restricted maximum likelihood (REML) using the asreml-R package (Butler et al. 2009). For the computation of the likelihood $R^{2}$, we used the R package nlme (Pinheiro et al. 2017). The results of the f-CSRT models were obtained using the Wald statistics as described in S6. All procedures in this study have been compiled in R packages and are available in the repository (https://github.com/vincentgarin/MPP_EUNAM/software/mppR_1.0.tar.gz).

### Cross validation

We adapted the CV procedure described by Utz et al. (2000) to the MPP context. For each of the 48 combinations of QTL model and scenario, we performed 100 CV runs by replicating 20 times a fivefold CV procedure. One run of CV was composed of the following steps: (1) the full data set was partitioned at within-cross level into a training set (TS) and a validation set (VS); (2) QTL detection was performed using the TS and the proportion of genetic variance explained in the TS was computed by $\mathrm{pTS} = R_{\mathrm{adj.TS}}^{2}/h^{2}$; and (3) the proportion of genetic variance predicted in the VS was calculated by $\mathrm{pVS} = \mathrm{cor}(\varvec{y_{\mathrm{VS}}}, \varvec{\hat{y}_{\mathrm{VS}}})/h^{2}$, representing the Pearson correlation between the observed values ($\varvec{y_{\mathrm{VS}}}$) and the predicted values ($\varvec{\hat{y}_{\mathrm{VS}}} = \varvec{X_{\mathrm{VS}}}\varvec{\hat{\beta }_{\mathrm{TS}}}$). The $\mathrm{pVS}$ were computed within crosses. An estimate at the full MPP level was obtained by taking a weighted average of the within cross values ($\mathrm{p\bar{V}S}$) accounting for the cross sizes. We evaluated the relative bias of a model by looking at the difference between ($\mathrm{pTS}$) and ($\mathrm{p\bar{V}}S$).

To reduce the computational time for CV, we thinned the set of markers by selecting the most polymorphic marker at every 1, 1.05, and 1.05 centi-Morgan for the long, heterogeneous, and short subsets, respectively. For each CV scenario, we determined the significance threshold running 1000 permutations on the full data set. The threshold of the MQE model was again determined by averaging the values obtained for the parental, ancestral, and bi-allelic models. For the CV procedure, we used the f-CSRT approximation for threshold computation and QTL detection in all scenarios using cross-specific residual terms.

## Results

### Subset properties

For both traits, DMY and PH, the estimated genetic variance per cross tended to increase when the genetic relatedness between the peripheral and the central parent decreased (Table 1; supplemental Figure S8). However, as in other studies (e.g., Hung et al. 2012), this relationship was not significant. The degrees of relatedness based on the allele clustering from clusthaplo resulted in 4.02, 4.09, and 4.56 ancestral alleles on average for the short, heterogeneous, and long subsets, respectively. This means that the difference in diversity between subsets was not high. The $-\mathrm{log}_{10}(p$ value) significance thresholds that were computed increased according to our expectation from the parental to the bi-allelic model, probably due to the reduction of test degrees of freedom (supplemental Table S9).

### Full subsets’ QTL detection

**Fig. 2:**
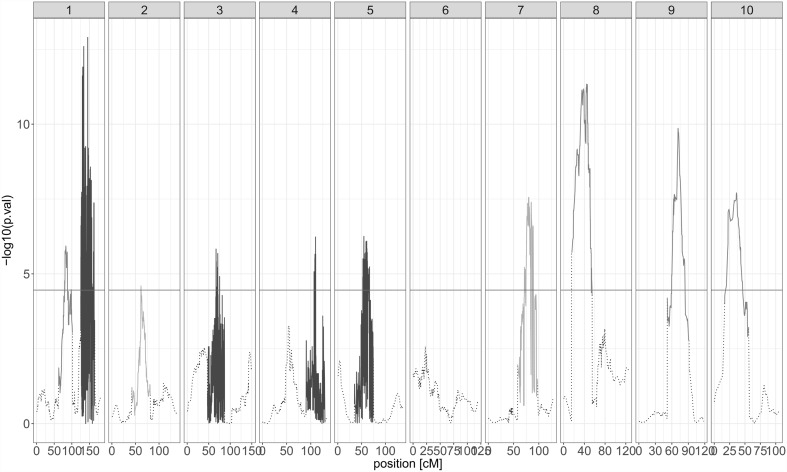
Example of MQE QTL profile result for PH in the heterogeneous subset. The *colours* drawn 40 cM around the detected positions represent the type of QTL effect at that locus (*red* parental, *green* ancestral, and *blue* bi-allelic) (colour figure online)

Table 2 presents the results of QTL detection using the full (non-partitioned) subsets for the different combinations of QTL effect and type of residual variance. For the type of QTL effect, we noticed that for DMY, the QTL detection results are similar in the short subset across the different models. In the heterogeneous and long subsets, however, the more parsimonious models (ancestral and bi-allelic) detected more QTLs and explained a larger percentage of genetic variation. For PH, this tendency was inversed. For example, the parental CSRT model explained 50.4% of the genetic variance, while the ancestral and bi-allelic models explained 40.1 and 38.3%, respectively. The MQE models detected more QTLs and explained a larger part of the genetic variance (see also Fig. 2). This was especially true for the MQE CSRT model, because, except for PH in the heterogeneous subset, it explained the largest part of the genetic variance. Concerning the residual term, the results of the HRT and CSRT models were similar for DMY. For PH, we could observe, based on the explained genetic variance, that the CSRT models generally outperformed the HRT models.

**Table 2:** QTL detection results of the full subsets analyses (short, heterogeneous, and long) per trait (DMY, PH) for the different QTL effects (parental, ancestral, bi-allelic, and MQE) and types of residual term (HRT, CSRT)

	DMY				PH			
	Parental	Ancestral	Bi-allelic	MQE	Parental	Ancestral	Bi-allelic	MQE
Short								
HRT	$3^{\mathrm{a}}$ $(20.6)^{\mathrm{b}}$	3 (20.6)	3 (18.7)	3 (1/2/–)$^{\mathrm{c}}$ (20.5)	7 (43.6)	6 (40.2)	7 (39.7)	6 (3/1/2) (41.6)
CSRT	3 (19.4)	4 (21.9)	4 (20.3)	4 (1/2/1) (22.6)	8 (50.4)	6 (40.1)	8 (38.3)	9 (5/1/3) (52)
Het.								
HRT	–	3 (13.2)	3 (15.3)	3 (1/–/2) (18.5)	7 (46.5)	9 (47.9)	6 (39.1)	10 (4/2/4) (55.3)
CSRT	1 (8.9)	3 (15.4)	3 (14.5)	4 (1/1/2) (20.8)	10 (57.3)	11 (58)	8 (46.6)	9 (3/3/3) (53.1)
Long								
HRT	2 (11.3)	1 (5.9)	5 (22.2)	7 (1/–/6) (32)	8 (42.8)	7 (38.7)	8 (38.5)	5 (1/3/1) (35.3)
CSRT	2 (10.4)	2 (9.1)	5 (22.1)	8 (3/–/5) (37)	8 (43.5)	8 (43)	9 (38.8)	10 (1/4/5) (49.3)

$^\mathrm{a}$ Number of detected QTLs. – for no QTL detected

$^\mathrm{b}$ Global adjusted $R^{2}$ in $\%$

$^\mathrm{c}$ Number of detected QTLs per incidence matrix type (parental/ancestral/bi-allelic)

### Cross validation

**Fig. 3:**
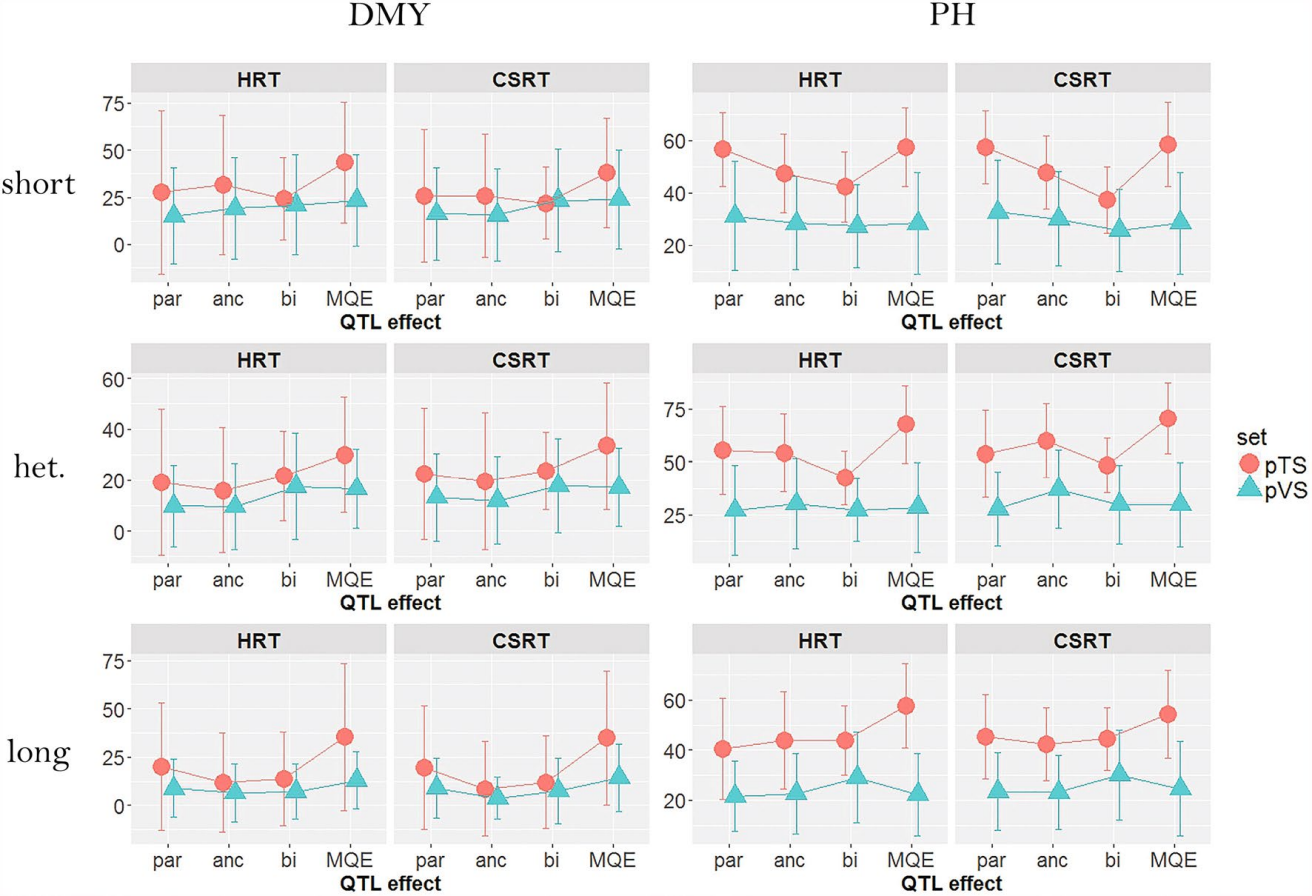
Cross-validation results over 100 runs. Average proportion of explained and predicted genetic variance (±2 $\times$ standard deviation) in the training and validation sets for each combination of trait (DMY and PH), subset (short, heterogeneous, and long), type of QTL effect (parental, ancestral, bi-allelic, and MQE), and residual term (HRT and CSRT)

The plots in Fig. 3 contain the CV results and show the similarity between the HRT and CSRT models. Furthermore, the MQE model had the largest pTS in all configurations. The MQE pVS was also the highest or equal to the highest single type of QTL effect model for DMY but not for PH. For PH, the different types of QTL effect model tend to give similar results in terms of pVS.

In the short subset for DMY, we observe that pVS increased with the parsimony of the model, since the ancestral and bi-allelic models obtained larger scores. For PH in the same subset, the results were opposite with the parental model having a larger average pVS. In the long subset, for DMY, we could not notice any difference in terms of pVS between the parental, ancestral, and bi-allelic models. For PH, however, the bi-allelic model performed better.

A final noteworthy result is that the difference between pTS and pVS (bias) was often reduced for the more parsimonious models especially for the bi-allelic model. This is, for example, the case in the short subset for both traits.

## Discussion

### Subset properties

The results of parental clustering of the different subsets showed that the genetic diversity was high in the three subsets, because the average clustering results showed a number of ancestral alleles that was close to the theoretical maximum number of six, being the number of parental alleles. This is consistent with the fact that this maize population was designed to reflect Northern European genetic diversity. The limited amount of difference between the subsets can be explained by the relatively reduced range of genetic similarities across the crosses as reflected in *SM* coefficients between the parents of the crosses (Table 1).

### Model performance given population diversity and type of QTL effect

Our first proposition implied that the relative performance of QTL models in terms of QTL detection would increase with model parsimony when the MPPs are derived from genetically close parents. The underlying reasoning is that higher genetic proximity allows reducing the number of parameters needed to model the QTL effect, producing a gain in power for QTL detection. We expected that the pVS would increase with model parsimony in the short subset. On the other hand, in the long subset, models with a higher number of parameters, such as the parental model, would help to model the assumed increased diversity and give better results in terms of pVS.

We did not observe these trends in the CV results (Fig. 3). The increase of pVS from the parental to bi-allelic models in the short subset for DMY is conform to our expectation. However, for PH in the short subset, we observed the opposite trend with pVS decreasing with model parsimony. In the long subset, we expected that the pVS would decrease with more parsimonious models. However, for PH, we noticed that the bi-allelic model gave the best result. Various reasons can be mentioned to explain that we did not observe the pattern expected from proposition 1. The first is that the difference in genetic diversity between the short and long subsets was not pronounced enough to have different QTL effects’ models performing differently. A second reason is that increased genetic distance between parents will not automatically translate into increased genetic variance. Third, the sample sizes may have been too small to show the expected patterns with not enough QTLs being detected and insufficient power to distinguish between QTL effect models and between homogeneous and heterogeneous residual genetic variances.

An important result was that in three scenarios out of six (short DMY, heterogeneous DMY, and long PH), the bi-allelic model gave the largest pVS. From a general point of view, more parsimonious models, especially the bi-allelic model, gave results with a reduced bias (difference between pTS and pVS). The improvement of QTL detection in MPP using more parsimonious models based on shared polymorphism has been an important topic in MPP QTL analysis. While simulation studies have supported this idea (Rebaï and Goffinet 1993; Jansen et al. 2003; Leroux et al. 2014), only few real data analyses have confirmed it (Blanc et al. 2006; Bardol et al. 2013). Of course, the superiority of the bi-allelic model depends on the sample size. With larger sample sizes, the bi-allelic model will be more often inferior to more complex QTL effect models.

Other real data analyses did not support the idea that a power gain could be achieved by integrating relatedness between crosses or parents into the analysis (Li et al. 2005; Coles et al. 2010; Steinhoff et al. 2011, 2012; Liu et al. 2012; Würschum et al. 2012; Giraud et al. 2014). In all these studies, the reference cross or parent specific model yielded the best results. For the full subset analysis of PH (Table 2), we also noticed that the parental model explained a higher proportion of the genetic variation. Cross or parent specificity of the QTL effects seems, therefore, to be important in MPP QTL mapping. Several factors can explain the presence of complex allelic series in an MPP context: (1) multiple alleles; (2) different allele frequencies per cross; (3) difference of linkage disequilibrium between markers and QTLs per cross; (4) cross-specific dominance ratio; and/or (5) interaction with the genetic background (Steinhoff et al. 2012; Blanc et al. 2006). For example, the simulation study of Li et al. (2016) demonstrated that the parental model outperformed the bi-allelic model only in case of strong interaction between the QTL and the genetic background.

Many of these studies did, however, not use CV to validate their results (e.g., Coles et al. 2010). In the paper of Han et al. (2016), CV was used to evaluate the model performance and no differences were found in terms of bias between the different tested models. Other authors using CV, such as Liu et al. (2012) and Würschum et al. (2012), did find a reduced bias for the bi-allelic model with respect to the cross-specific model, as we did. For the reduction of bias by more parsimonious models, statistical and biological arguments can be formulated.

From a statistical perspective, the inclusion of multiple QTL effects, such as in the parental model, will make the procedure susceptible to overfitting (Friedman et al. 2001). Therefore, parental effects may model variation that is specific to the TS but that will not necessarily be typical for the VS. Genetically, it has been shown that an important part of the polymorphic variation in maize was cross or even genotype specific (Myles et al. 2009). In contrast, the bi-allelic model contains just two parameters and assumes that these are present across the whole MPP, and so will be shared between TS and VS.

From a biological perspective, we interpret the parental model as being based on more recent sources of relatedness, whereas the bi-allelic model is closer to the original mutation (Powell et al. 2010). Since SNPs represent older polymorphisms, they tend to be better distributed across the whole population (Nicholson et al. 2002; Speed and Balding 2015). Therefore, QTLs detected with the bi-allelic model may be better spread throughout the whole population and be more easily transmitted to the next generation.

### Multi-QTL effect (MQE) model

As mentioned in the result section, the MQE model performed better in terms of pTS than the models assuming a single type of QTL effect along the genome (Fig. 3). The larger proportion of genetic variance explained in the TS by the MQE model in comparison with the VS may be explained by the greedy forward regression strategy used to build the MQE model. Indeed, this strategy includes many genome scans as at each QTL detection step, a genome-wide scan is performed for each type of QTL effect conditional on all earlier identified QTLs. The increased number of scans can lead to an overfit to the data present in the TS, while the variation modelled in the TS is not necessarily typical of the VS, as we noticed for the trait PH. However, for DMY, the MQE method did obtain larger pVS, which supports our second proposition that the inclusion of different types of QTL effects in the same model can lead to a better description of the phenotypic variation.

The MQE model seems, therefore, to be a useful strategy to model phenotypic variations in MPPs. The MQE model is aims at finding the most adequate type of QTL effect for each QTL position. In philosophy, it is similar to a Bayesian approach proposed by Jannink and Wu (2003) who treated the number of alleles at a QTL position as a random parameter. The MQE model is probably computationally less demanding. It may require improvements on the correction for multiple testing to avoid overfitting by taking into account the number of scans performed. Alternatively, it may be better to take the maximum observed threshold across the three threshold values corresponding to the three types of QTL effects in place of the mean threshold, as we did now.

### Model performance under different assumptions for the residual term

Our Proposition 3 implied that heterogeneity of genetic distance between the central and the peripheral parent would require an elaborate model to accommodate the heterogeneity of variance for the polygenic effects. Cross-specific residual terms should give an improved description of the residual genetic variance against which to test for the QTL effects in comparison with a simpler model based on a single residual variance component (supplemental file S5). We expected the difference between the HRT and CSRT models to be largest in the heterogeneous subset. The CV results (Fig. 3) did not show any difference between the HRT and CSRT models, maybe due to too small population sizes. In the full subset analysis (Table 2), we could, however, notice that in several cases, the CSRT model outperformed the HRT model. For example, the MQE CSRT model explained a larger proportion of genetic variance than the MQE HRT model in five scenarios out of six.

The absence of difference between the HRT and CSRT models for the CV results can be caused by the use of the f-CSRT approximation. Alternatively, the sample sizes for the crosses may have been too small to allow HRT and CSRT to be tested as being different. The f-CSRT seems to have less QTL detection power than the exact solution, because the correction for heterogeneous residual term only reflects the general level of heterogeneity. In the exact solution, however, the residual genetic variance is calculated at each genomic position conditional on the estimated QTL effects. In that case, the Wald statistics can truly benefit from the within cross-variance reductions following from the included QTL effects. Therefore, given the full subsets results (Table 2), we still consider that the CSRT model can improve QTL detection in MPP.

## Perspective

The result presented in this study illustrates the potential of using different types of QTL effect models and also represents different ways to model genetic relatedness in an MPP. We demonstrated that it can be interesting to integrate multiple assumptions about the origin of the QTLs in the same multi-locus QTL model. The question of genetic relatedness definition is one of the most important ones in genetics (Fisher 1918). We know that statistical dependence between haplotypes is the result of a complex evolutionary/selection process, where mutation, recombination, and coalescence of lineage act jointly (Rosenberg and Nordborg 2002). We think that the use of relatedness and shared polymorphisms, through a better modelling of genetic relationships, can improve QTL detection in MPPs.

A first way to improve the estimation of genetic relatedness modelling is using SNP markers in place of pedigree information. According to Powell et al. (2010), this represents a more unified way to measure relatedness and allows to solve the apparent conflict between IBD and IBS methods, because when marker density is high, the different categories of ancestors merge. A genetic relationship matrix (GRM) has been widely used in GWAS analysis to control for population structure and/or model polygenic effect within mixed models (Yu et al. 2006; Malosetti et al. 2007). This technique was also employed with success to estimate genetic effects (Yang et al. 2010; Speed et al. 2012). The extension of such a methodology to MPP QTL detection represents a promising option.

A second, more challenging option is to use methods for IBD computation using ancestral lines higher up in the MPP pedigree as a reference like in Zheng et al. (2015). Finally, Bayesian methods have also great potential to deal with complex pedigrees. In this framework, efforts have been made to model more appropriately the relationships between lines. For example, Jannink and Wu (2003) proposed to treat the number of ancestral alleles as a random parameter in an attempt to estimate the most probable relatedness scheme between MPP parents. In the same vein, Ter Braak et al. (2010) developed an algorithm to infer latent ancestral class origins of population’s founders allowing to sample parent origin in a Bayesian context.

From a general point of view, we would like to emphasize the main motivation for our QTL mapping approach: try to make as explicit as possible the connection between the biological assumptions and the properties of the statistical model that is used. A constant dialogue between these two dimensions is certainly a promising way to make progress in both the understanding of biological processes occurring in MPPs and their statistical modelling.

### Author contribution statement

 All authors developed the theoretical framework and the hypotheses, edited the manuscript, and read and approved the final version. VG wrote the software used for data analysis, analysed data, and wrote the manuscript. VW reviewed the software. FvE coordinated the research.

## Electronic supplementary material

Below is the link to the electronic supplementary material.

Supplementary material 1 (PDF 1351 kb)
