# Supplementary material for: How do the type of QTL effect and the form of the residual term influence QTL detection in multi-parent populations? A case study in the maize EU-NAM population
Source: Theor Appl Genet. 2017 May 25;130(8):1753–64. doi: 10.1007/s00122-017-2923-3 (PMC5511610; doi:10.1007/s00122-017-2923-3)
Supplement: Supplementary file 1 — Supplementary material 1 (PDF 1351 kb) [file 122_2017_2923_MOESM1_ESM.pdf]

Theoretical and Applied Genetics manuscript No.
(will be inserted by the editor)

How do the type of QTL effect and the form of the residual term influence QTL detection in multi-parent populations? A case study in the maize EU-NAM population - Supplementary material

Vincent Garin · Valentin Wimmer ·
Sofiane Mezmouk · Marcos Malosetti ·
Fred van Eeuwijk

the date of receipt and acceptance should be inserted later

Vincent Garin
Biometris, Wageningen University and research Center, P.O Box 100, 6700AC Wageningen, The Netherlands.
Tel.: 0031 317 83 574
E-mail: vincent.garin@wur.nl

Valentin Wimmer
KWS SAAT SE, Einbeck, Germany

S1: Nei and Li genotype similarity coefficient

$GS = 2N_{ij}/(N_i + N_j)$ where N_{ij} is the number of common sites between i and j and $N_{i(j)}$ is the total number of observed sites in individual $i(j)$ (Nei and Li, 1979). In our situation, considering the same number of SNPs in parent i and j makes GS equivalent to the simple matching coefficient (SM).

S2: Simple matching coefficients table between parents of the EU-NAM Dent panel

	B73	D06	D09	EC169	F252	F353	F618	Mo17	UH250	UH304	W117
B73	1	0.566	0.556	0.685	0.561	0.557	0.59	0.554	0.579	0.584	0.542
D06		1	0.866	0.643	0.611	0.586	0.599	0.559	0.836	0.624	0.568
D09			1	0.585	0.628	0.618	0.589	0.559	0.762	0.648	0.564
EC169				1	0.556	0.558	0.589	0.558	0.613	0.591	0.538
F252					1	0.633	0.578	0.576	0.609	0.648	0.579
F353						1	0.589	0.567	0.575	0.761	0.565
F618							1	0.552	0.595	0.606	0.559
Mo17								1	0.578	0.586	0.564
UH250									1	0.614	0.563
UH304										1	0.587
W117											1

Average genetic similarity score per subset

$$\bar{SM}_{short} = 0.639$$
$$\bar{SM}_{het.} = 0.613$$
$$\bar{SM}_{long} = 0.573$$

S3: Principal component bi-plot of the EU-NAM Dent parents

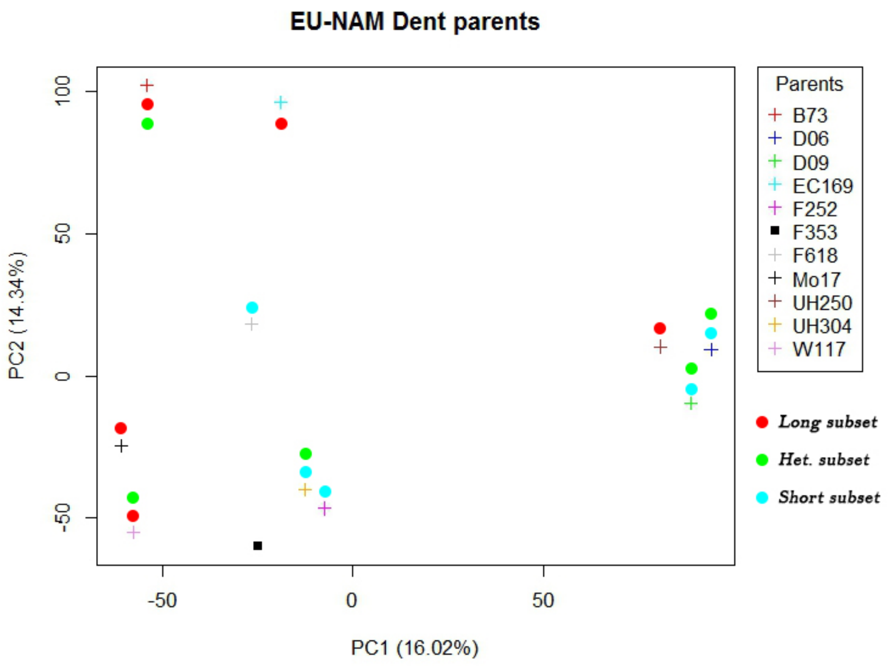

S4: Genetic marker map of the different subsets

Short subset

Chromosome	N	Length(cM)	Average spacing	maximum spacing
1	985	183.8	0.2	1.8
2	633	137.9	0.2	2.0
3	702	150.9	0.2	2.4
4	617	133.9	0.2	2.2
5	563	136.5	0.2	3.8
6	482	119.9	0.2	3.1
7	470	128.9	0.3	3.3
8	496	125.6	0.3	2.6
9	449	118.4	0.3	2.4
10	340	105.8	0.3	5.3
Overal	5737	1341.6	0.2	5.3

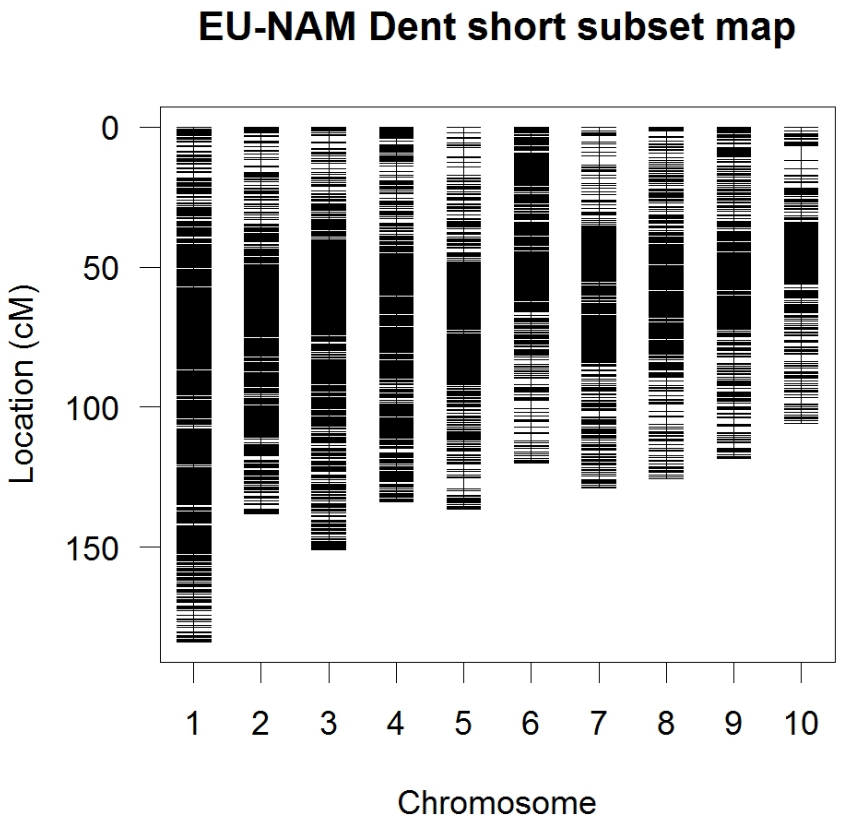

Heterogeneous subset

Chromosome	N	Length(cM)	Average spacing	maximum spacing
1	1055	184.5	0.2	1.9
2	630	137.9	0.2	2.1
3	711	151.0	0.2	2.9
4	620	134.6	0.2	2.2
5	573	136.6	0.2	3.8
6	526	119.9	0.2	2.6
7	493	128.9	0.3	2.6
8	527	125.6	0.2	2.9
9	467	118.4	0.3	2.4
10	332	105.8	0.3	7.3
Overal	5934	1343.2	0.2	7.3

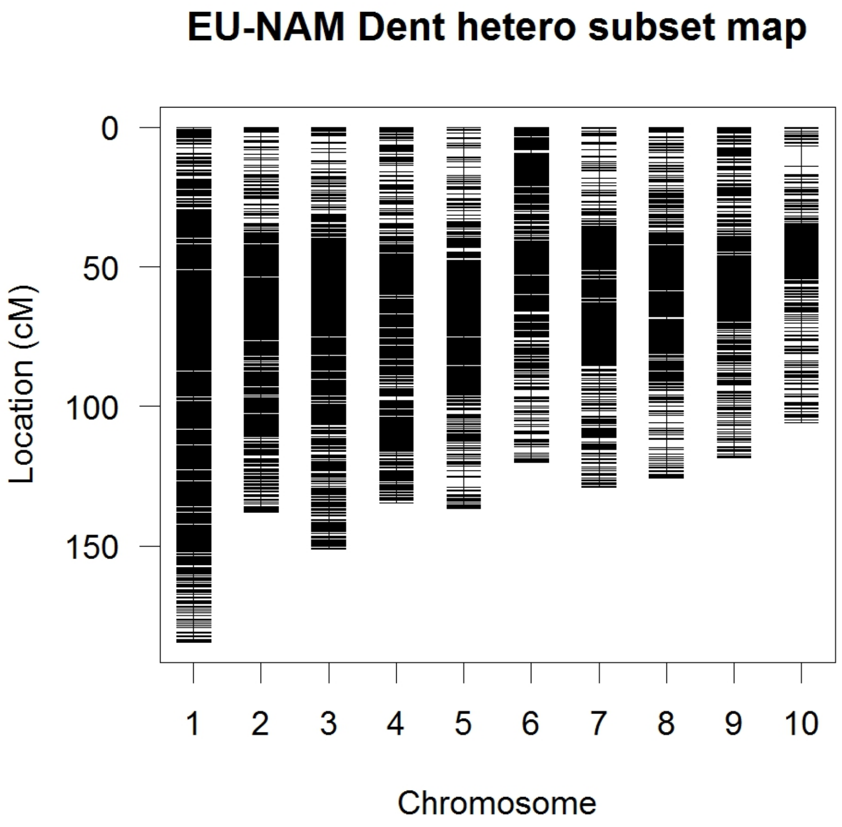

Long subset

Chromosome	N	Length(cM)	Average spacing	maximum spacing
1	1121	184.5	0.2	1.9
2	641	137.9	0.2	2.0
3	743	151.0	0.2	1.9
4	647	134.6	0.2	3.1
5	598	136.6	0.2	4.1
6	550	119.9	0.2	2.1
7	524	128.9	0.2	2.2
8	560	125.6	0.2	2.9
9	484	118.4	0.2	2.7
10	344	105.8	0.3	5.7
Overal	6212	1343.2	0.2	5.7

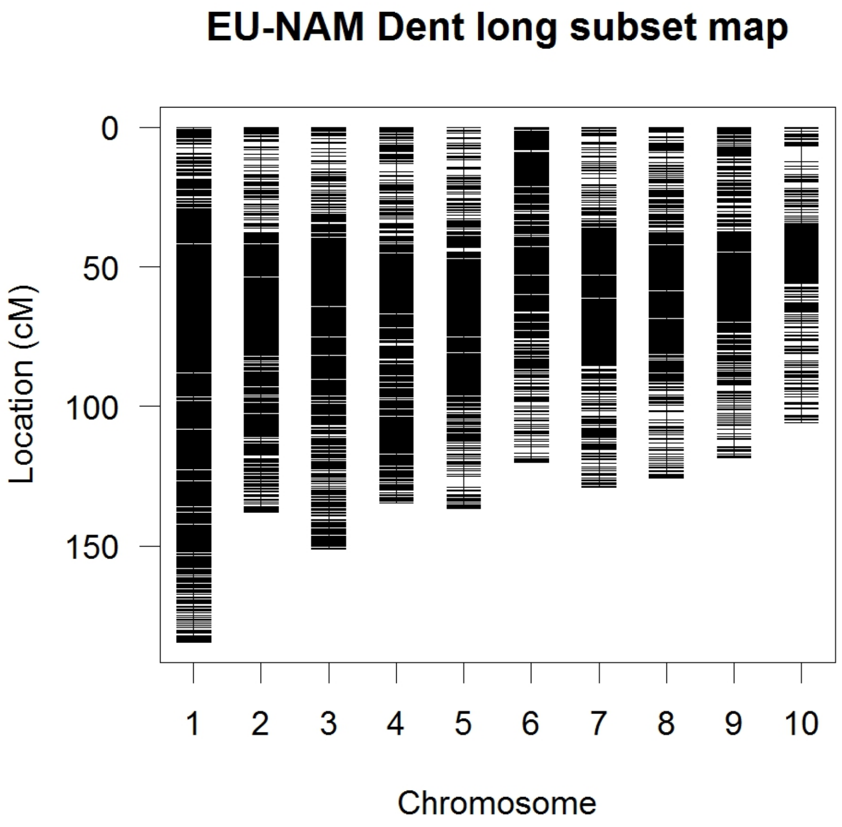

S5: Test statistic of the QTL effect

Wald test derivation

The significance of the estimated QTL effects $\hat{\boldsymbol{\beta}}_{\boldsymbol{Q}}$ can be estimated using the Wald test (Wald, 1943). From model (2), the phenotype values $\boldsymbol{y}$ have an expectation equivalent to $\boldsymbol{X\beta}$ and their variance is $\boldsymbol{R}$ (McCulloch and Searle, 2001, 6.5, 6.6). In such a situation, we can derive a generalized estimate for $\boldsymbol{\beta}$ and its variance as follow (Rao et al, 2008, 4.65, 4.66):

$$\hat{\boldsymbol{\beta}} = (\boldsymbol{X'R}^{-1}\boldsymbol{X})^{-1}\boldsymbol{X'R}^{-1}\boldsymbol{y} \tag{1}$$

$$V(\hat{\boldsymbol{\beta}}) = (\boldsymbol{X'R}^{-1}\boldsymbol{X})^{-1} \tag{2}$$

In its general form, the Wald statistic is equal to (McCulloch and Searle, 2001, 5.39):

$$W = (\hat{\boldsymbol{\beta}} - \hat{\boldsymbol{\beta}}_{\boldsymbol{0}})'[V(\hat{\boldsymbol{\beta}})]^{-1}(\hat{\boldsymbol{\beta}} - \hat{\boldsymbol{\beta}}_{\boldsymbol{0}}) \tag{3}$$

Under the null hypothesis we assume $\hat{\boldsymbol{\beta}} = \hat{\boldsymbol{\beta}}_{\boldsymbol{0}} = [0\ 0 \ldots 0]'$ After substituting (1) and (2) in (3), we can rewrite the Wald statistic like that:

$$\begin{aligned} W &= \boldsymbol{y}'\hat{\boldsymbol{R}}^{-1}\boldsymbol{X}(\boldsymbol{X}'\hat{\boldsymbol{R}}^{-1}\boldsymbol{X})^{-1}\boldsymbol{X}'\hat{\boldsymbol{R}}^{-1}\boldsymbol{y} \\ &= \boldsymbol{y}'\hat{\boldsymbol{R}}^{-1}\boldsymbol{X}\hat{\boldsymbol{\beta}} \\ &= \boldsymbol{y}'\hat{\boldsymbol{R}}^{-1}\boldsymbol{Hy} \\ &= \boldsymbol{y}'\hat{\boldsymbol{R}}^{-1}\hat{\boldsymbol{y}} \end{aligned} \tag{4}$$

where,

$$\boldsymbol{H} = \boldsymbol{X}(\boldsymbol{X}'\hat{\boldsymbol{R}}^{-1}\boldsymbol{X})^{-1}\boldsymbol{X}'\hat{\boldsymbol{R}}^{-1}$$

is the generalized hat matrix.

The previous expression W represent a global Wald test coefficient $W(\hat{\boldsymbol{\beta}})$ including both effect of the cross intercepts $\hat{\boldsymbol{\beta}}_{\boldsymbol{c}}$ and the QTL effects $\hat{\boldsymbol{\beta}}_{\boldsymbol{Q}}$. The significance of the QTL effects $W(\hat{\boldsymbol{\beta}}_{\boldsymbol{Q}})$ can be obtained doing the difference between $W(\hat{\boldsymbol{\beta}})$ and $W(\hat{\boldsymbol{\beta}}_{\boldsymbol{c}})$, the Wald statistic of a model including only the cross intercept terms.

$$W(\hat{\boldsymbol{\beta}}_{\boldsymbol{Q}}) = W(\hat{\boldsymbol{\beta}}) - W(\hat{\boldsymbol{\beta}}_{\boldsymbol{c}}) \tag{5}$$

$W(\hat{\boldsymbol{\beta}}_{\boldsymbol{Q}})$ is therefore proportional to $W(\hat{\boldsymbol{\beta}})$ described in (4). $W(\hat{\boldsymbol{\beta}}_{\boldsymbol{Q}})$ tests the hypothesis of all QTL effects equal zero versus at least on component of $\hat{\boldsymbol{\beta}}_{\boldsymbol{Q}}$ being non null. $W(\hat{\boldsymbol{\beta}}_{\boldsymbol{Q}})$ follows a χ^2 distribution with degree of freedom equal to the rank of $\boldsymbol{X}_{\boldsymbol{Q}}$ (the number of estimated QTL effects).

Interpretation

Expression (4) allows to summarise the main features of the proposed QTL models: parsimony versus goodness of fit and accurate form of the residual term.

Parsimony vs goodness of fit

Since the degree of freedom of the Wald test depends on the number of estimated parameters, more parsimonious models like the ancestral or the bi-allelic model will automatically increase the significance level of $W(\hat{\boldsymbol{\beta}}_{\boldsymbol{Q}})$. The use of more parsimonious models is however not always a guaranty of better results (Bardol et al, 2013; Steinhoff et al, 2011). An important criteria to be balanced with parsimony is the necessity to infer allele effects that capture correctly the trait variability. To illustrate this, we can further reduce expression $W(\hat{\boldsymbol{\beta}})$ (4) and substitute it in (5) to draw the following relationship:

$$W(\hat{\boldsymbol{\beta}}_{\boldsymbol{Q}}) \propto \boldsymbol{y}'\hat{\boldsymbol{y}} = \sum_{n=1}^{N} y_i \hat{y}_n \tag{6}$$

From this expression we can see that the more the vector $\boldsymbol{y}$ and the vector $\hat{\boldsymbol{y}}$ vary in the same direction the higher will be $W(\hat{\boldsymbol{\beta}}_{\boldsymbol{Q}})$. This simply means that the form of the QTL incidence matrix $\boldsymbol{X_Q}$ should be chosen to give genetic estimates $\hat{\boldsymbol{\beta}}_{\boldsymbol{Q}}$ allowing a projection $\hat{\boldsymbol{y}} = \boldsymbol{Hy}$ capturing the highest proportion of the trait variability. If these variations are due to parental or cross-specific effects, corresponding genetic effect estimates $\hat{\boldsymbol{\beta}}_{\boldsymbol{Q}}$ capturing these local variation should perform better at the price of a higher number of parameter to estimate. On the other hand if the effects are similar through the MPP, a reduced number of parameters will capture this variability and allows gains in power by a lower number of degree of freedom.

Accurate form of the residual term

The test statistic is also influenced by the chosen VCOV. As we can see in expression (4) each element composing $W(\hat{\boldsymbol{\beta}}_{\boldsymbol{Q}})$ is weighted by the estimated $\hat{\boldsymbol{R}}$. The more $\hat{\boldsymbol{R}}$ will reflect the correct form of the residual term the more accurate will be the QTL detection process. In the HRT case, substituting (4) in (5), we can write the following relationship:

$$W(\hat{\boldsymbol{\beta}}_{\boldsymbol{Q}}) \propto \sum_{n=1}^{N} \frac{y_n \hat{y}_n}{\sigma_r^2} \tag{7}$$

In this situation each elements is weighted by an average level of uncertainty σ_r^2 which may not be representative of crosses particularities. In the CSRT situation, we have:

$$W(\hat{\boldsymbol{\beta}}_{\boldsymbol{Q}}) \propto \sum_{c=1}^{n_c} \sum_{n=1}^{N_c} \frac{y_n \hat{y}_n}{\sigma_{r_c}^2} \tag{8}$$

Here, the different elements will be weighted by cross-specific variance residual terms $\sigma_{r_c}^2$ that take into account the potential differences of uncertainty between crosses. The more $\sigma_{r_c}^2$ are heterogeneous the more a CSRT model is needed to

handle this variability. In such case, a HRT model will tend to be more liberal when uncertainty is in fact larger (cross 1 in Table 1) and more conservative when effects are in reality more certain (cross 3 in Table 1).

Table 1 Illustration of the difference between the HRT and CSRT assumption in an heterogeneous MPP and its effect on the QTL test statistic ($\frac{\beta}{\sigma^2}$).

	$\sigma^2_{r_c}$ ("True")	σ^2_r ("Average")	Test (HRT)		Test (CSRT)
cross 1	190		$\frac{\beta_1}{100}$	$>$	$\frac{\beta_1}{190}$
cross 2	100	100	$\frac{\beta_2}{100}$	$=$	$\frac{\beta_2}{100}$
cross 3	10		$\frac{\beta_3}{100}$	$<$	$\frac{\beta_3}{10}$

S6: CSRT model approximation

The idea is to first estimate the variance covariance structure $\hat{\boldsymbol{R}}$ and then use it in a generalized estimate of the Wald test (4):

$$W(\hat{\boldsymbol{\beta}}) = \boldsymbol{y}'\hat{\boldsymbol{R}}^{-1}\boldsymbol{X}(\boldsymbol{X}'\hat{\boldsymbol{R}}^{-1}\boldsymbol{X})^{-1}\boldsymbol{X}'\hat{\boldsymbol{R}}^{-1}\boldsymbol{y} \tag{9}$$

Since in the CSRT model $\hat{\boldsymbol{R}}$ contains only diagonal elements we can simply invert $\hat{\boldsymbol{R}}$ by doing $\hat{\boldsymbol{R}}^{-1} = 1/\hat{\boldsymbol{R}}$.

SIM

$$\text{Cr(Int) model:} \qquad \boldsymbol{y} = \boldsymbol{X_c}\boldsymbol{\beta_c} + \boldsymbol{r} \tag{10}$$

$$\text{Cr(Int) + Q model:} \qquad \boldsymbol{y} = \boldsymbol{X_c}\boldsymbol{\beta_c} + \boldsymbol{X_Q}\boldsymbol{\beta_Q} + \boldsymbol{r} \tag{11}$$

To estimate the significance of the QTL effect we can use the following incremental Wald statistics

$$W(\hat{\boldsymbol{\beta}}_{\boldsymbol{Q}}) = W([\hat{\boldsymbol{\beta}}_{\boldsymbol{c}}|\hat{\boldsymbol{\beta}}_{\boldsymbol{Q}}]) - W(\hat{\boldsymbol{\beta}}_{\boldsymbol{c}}) \tag{12}$$

The procedure to estimate the significance of the QTL effect genome-wide is the following:

1. Estimate $\hat{\boldsymbol{R}}$ from model (10).
2. Compute $W(\hat{\boldsymbol{\beta}}_{\boldsymbol{c}})$ substituting $\hat{\boldsymbol{R}}$ and $\boldsymbol{X} = \boldsymbol{X_c}$ in (9).
3. Compute at each position $W([\hat{\boldsymbol{\beta}}_{\boldsymbol{c}}|\hat{\boldsymbol{\beta}}_{\boldsymbol{Q}}])$ substituting $\hat{\boldsymbol{R}}$ and $\boldsymbol{X} = [\boldsymbol{X_c}|\boldsymbol{X_Q}]$ in (9).
4. Compute the p-value of the QTL effect using (12) and $W(\hat{\boldsymbol{\beta}}_{\boldsymbol{Q}}) \sim \chi^2_{df}$ with $df = Rank(\boldsymbol{X_Q})$.

CIM

$$\text{Cr(Int) + cof model:} \qquad \boldsymbol{y} = \boldsymbol{X_c \beta_c} + \boldsymbol{X_{cof} \beta_{cof}} + \boldsymbol{r} \tag{13}$$

$$\text{Cr(Int) + cof + Q model:} \qquad \boldsymbol{y} = \boldsymbol{X_c \beta_c} + \boldsymbol{X_{cof} \beta_{cof}} + \boldsymbol{X_Q \beta_Q} + \boldsymbol{r} \tag{14}$$

To estimate the significance of the QTL effect we can use the following incremental Wald statistics

$$W(\hat{\boldsymbol{\beta}}_{\boldsymbol{Q}}) = W([\hat{\boldsymbol{\beta}}_{\boldsymbol{c}} | \hat{\boldsymbol{\beta}}_{\boldsymbol{cof}} | \hat{\boldsymbol{\beta}}_{\boldsymbol{Q}}]) - W([\hat{\boldsymbol{\beta}}_{\boldsymbol{c}} | \hat{\boldsymbol{\beta}}_{\boldsymbol{cof}}]) \tag{15}$$

The procedure to estimate the significance of the QTL effect genome-wide is the following:

1. Estimate $\hat{\boldsymbol{R}}_{\boldsymbol{j}}$ for the different combinations of cofactor $\boldsymbol{X_{cof.j}}$ using model (13).
2. Compute $W([\hat{\boldsymbol{\beta}}_{\boldsymbol{c}} | \hat{\boldsymbol{\beta}}_{\boldsymbol{cof.j}}])$ for the different combinations of cofactor substituting $\hat{\boldsymbol{R}}_{\boldsymbol{j}}$ and $\boldsymbol{X} = [\boldsymbol{X_c} | \boldsymbol{X_{cof.j}}]$ in (9).
3. Compute at each position $W([\hat{\boldsymbol{\beta}}_{\boldsymbol{c}} | \hat{\boldsymbol{\beta}}_{\boldsymbol{cof.j}} | \hat{\boldsymbol{\beta}}_{\boldsymbol{Q}}])$ substituting $\hat{\boldsymbol{R}}_{\boldsymbol{j}}$ and $\boldsymbol{X} = [\boldsymbol{X_c} | \boldsymbol{X_{cof.j}} | \boldsymbol{X_Q}]$ in (9).
4. Compute the p-value of the QTL effect using (15) and $W(\hat{\boldsymbol{\beta}}_{\boldsymbol{Q}}) \sim \chi^2_{df}$ with $df = Rank(\boldsymbol{X_Q})$.

S7: Multi QTL effects model

The multi-QTL effect model uses a forward regression to build up a model in which different loci are allowed to have different types of QTL effects. At each step one QTL is added, and each of the different types of effects are compared. To select this position, we compute genome wide profiles using the types of QTL effects given by the user. Each of these profiles uses a single type of effect for all the tested (QTL) position.

$$\boldsymbol{y} = \boldsymbol{X_c \beta_c} + \boldsymbol{X_{Q1} \beta_{Q1}} + \boldsymbol{r} \tag{16}$$

Where, the QTL position $\boldsymbol{X_{Q1}\beta_Q}$ is parental, ancestral, or bi-allelic. We calculate therefore as many QTL profile as types of QTL effects chosen by the user.
From each of these profiles, the most significant position based on the -log10(p value) statistic is selected (e.g., $\boldsymbol{X_{Q1.par}}$, $\boldsymbol{X_{Q1.anc}}$, $\boldsymbol{X_{Q1.biall}}$). Note that the selected QTL positions might or not be at the same position. The one that increases the most the R^2_{adj} is selected as QTL. The selected position with its type of QTL effect is added to the cofactors list and the selection process starts again. If at step 1 we selected a bi-allelic QTL, then at step 2 the QTL profiles will be based on the following models:

$$\boldsymbol{y} = \boldsymbol{X_c \beta_c} + \boldsymbol{X_{q1.biall} \beta_{q1}} + \boldsymbol{X_{Q2} \beta_{Q2}} + \boldsymbol{r} \tag{17}$$

With again the tested QTL position $\boldsymbol{X_{Q2}\beta_{Q2}}$ taking, in each QTL profile, the form of one of the QTL effect specified by the user.

The procedure stops when there is no more significant position. The final list of QTL is tested simultaneously using a backward elimination. The final model could look like that:

$$\boldsymbol{y} = \boldsymbol{X_c \beta_c} + \boldsymbol{X_{q1.biall} \beta_{q1}} + ... + \boldsymbol{X_{q(t-1).par} \beta_{q(t-1)}} + \boldsymbol{X_{Qt.anc} \beta_{Qt}} + \boldsymbol{r} \tag{18}$$

S8: Genetic variance versus parental relatedness tables

The following graphs represent the scatter plots of estimated genetic variance per family on SM coefficient between the central parent and the peripheral one (see S1 and S2). The two trends represent the linear and quadratic trend respectively.

Dry matter yield

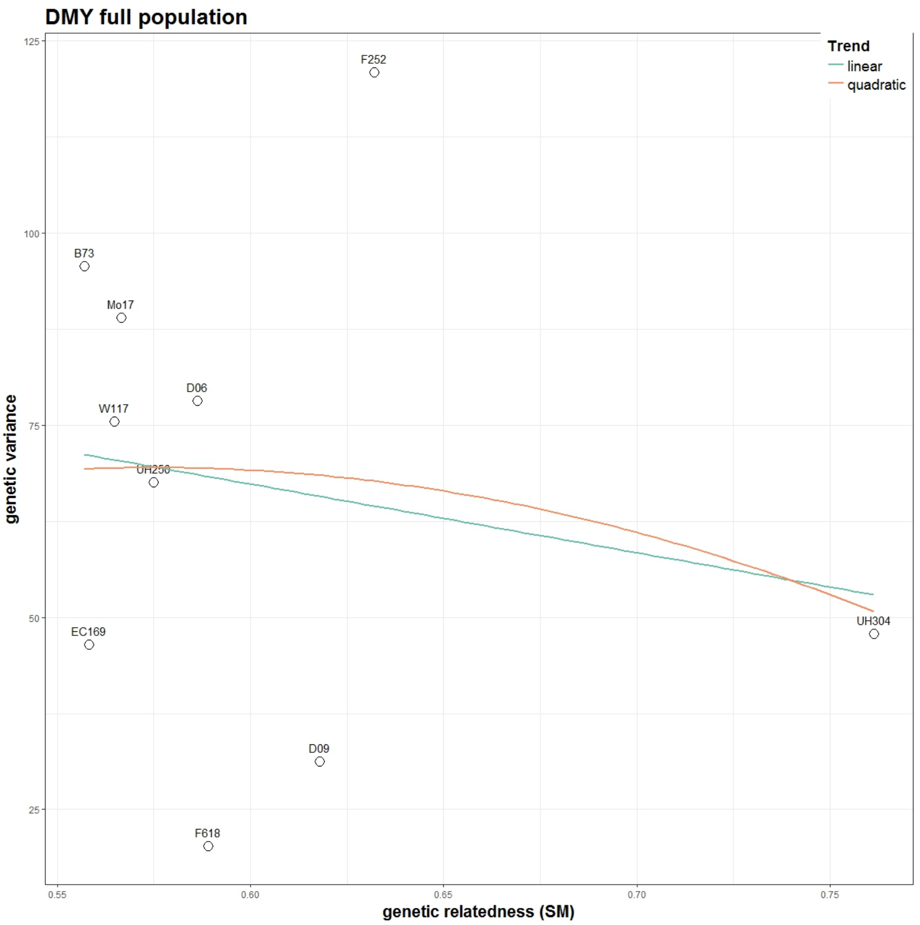

Plant height

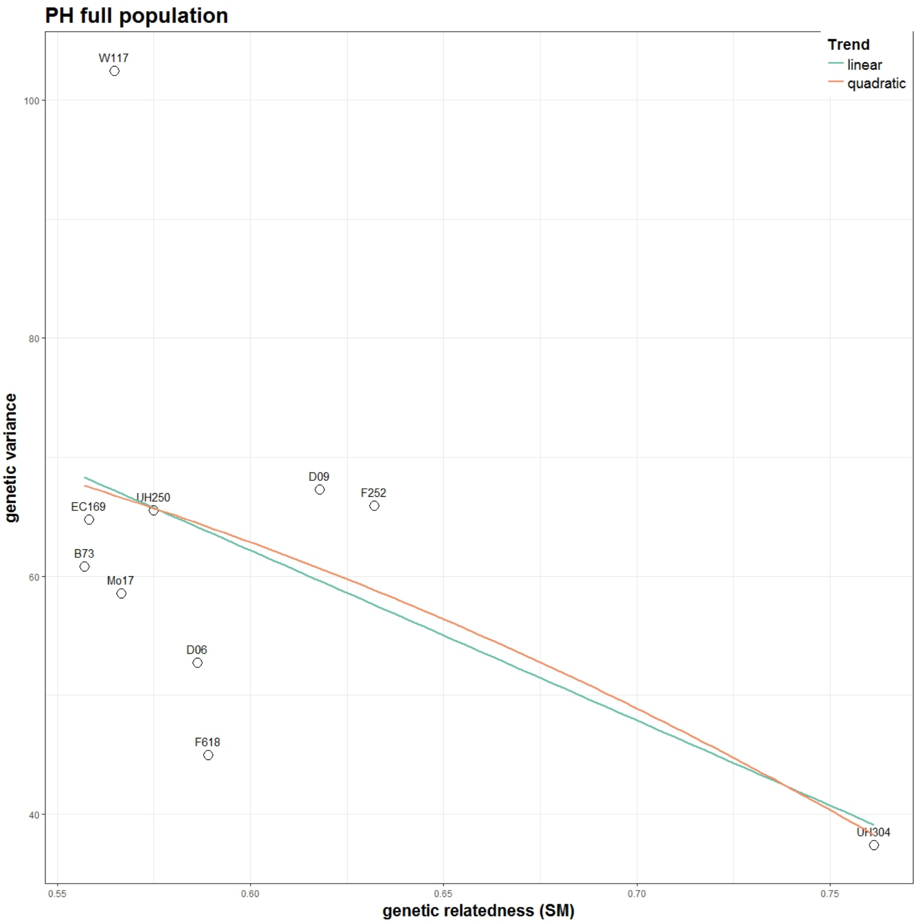

S9: Permutation threshold results

Significance thresholds determined by 1000 genome-wide permutations taking the -log10(p-value) of the upper 95% Wald statistic under the empirical null distribution as the critical value for rejection.

Dry mater yield

	parental	ancestal	bi-allelic	Average - MQE
short	3.89	4.09	4.66	4.21
het.	4.03	4.27	5.01	4.44
long	3.82	3.97	4.56	4.12
Average	3.91	4.11	4.74	

Plant height

	parental	ancestal	bi-allelic	Average - MQE
short	3.86	4.03	4.65	4.18
het.	3.97	4.22	5.18	4.46
long	3.77	4.14	4.85	4.25
Average	3.87	4.13	4.89	

References

Bardol N, Ventelon M, Mangin B, Jasson S, Loywick V, Couton F, Derue C, Blanchard P, Charcosset A, Moreau L (2013) Combined linkage and linkage disequilibrium qtl mapping in multiple families of maize (zea mays l.) line crosses highlights complementarities between models based on parental haplotype and single locus polymorphism. Theoretical and applied genetics 126(11):2717–2736

McCulloch CE, Searle SR (2001) Generalized, linear, and mixed models. Wiley Online Library

Nei M, Li WH (1979) Mathematical model for studying genetic variation in terms of restriction endonucleases. Proceedings of the National Academy of Sciences 76(10):5269–5273

Rao CR, Toutenburg H, Shalabh, Heumann C (2008) Linear Models and Generalizations: Least Squares and Alternatives. Springer

Steinhoff J, Liu W, Maurer HP, Würschum T, Friedrich C, Longin H, Ranc N, Reif JC (2011) Multiple-line cross quantitative trait locus mapping in european elite maize. Crop science 51(6):2505–2516

Wald A (1943) Tests of statistical hypotheses concerning several parameters when the number of observations is large. Transactions of the American Mathematical society 54(3):426–482
